# Association of HLA-B*41:02 with Henoch-Schönlein Purpura (IgA Vasculitis) in Spanish individuals irrespective of the HLA-DRB1 status

**DOI:** 10.1186/s13075-015-0622-5

**Published:** 2015-04-14

**Authors:** Raquel López-Mejías, Fernanda Genre, Belén Sevilla Pérez, Santos Castañeda, Norberto Ortego-Centeno, Javier Llorca, Begoña Ubilla, Sara Remuzgo-Martínez, Verónica Mijares, Trinitario Pina, Vanesa Calvo-Río, Ana Márquez, José A Miranda-Filloy, Antonio Navas Parejo, Marta Conde-Jaldón, Lourdes Ortiz-Fernández, Diego Argila, Maximiliano Aragües, Esteban Rubio, Manuel León Luque, Juan María Blanco-Madrigal, Eva Galíndez-Aguirregoikoa, Francisca González Escribano, J Gonzalo Ocejo-Vinyals, Javier Martín, Ricardo Blanco, Miguel A González-Gay

**Affiliations:** Epidemiology, Genetics and Atherosclerosis Research Group on Systemic Inflammatory Diseases, Rheumatology Division, Hospital Universitario Marqués de Valdecilla, IDIVAL, Santander, Spain; Medicine Department, Hospital Universitario San Cecilio, Granada, Spain; Rheumatology Department, Hospital Universitario La Princesa, IIS-Princesa, Madrid, Spain; Epidemiology and Computational Biology Department, School of Medicine, University of Cantabria, and CIBER Epidemiología y Salud Pública (CIBERESP), IDIVAL, Santander, Spain; Institute of Parasitology and Biomedicine López-Neyra (IPBLN-CSIC) and Systemic Autoimmune Diseases Unit, Hospital Clínico San Cecilio, Granada, Spain; Division of Rheumatology, Hospital Universitario Lucus Augusti, Lugo, Spain; Nephrology Department, Hospital Universitario San Cecilio, Granada, Spain; Immunology Department, Hospital Universitario Virgen del Rocío, Sevilla, Spain; Dermatology Department, IIS-IP, Hospital de la Princesa, Madrid, Spain; Rheumatology Department, Hospital Universitario Virgen del Rocío, Sevilla, Spain; Rheumatology Department, Hospital Universitario de Basurto, Bilbao, Spain; Immunology Department, Hospital Universitario Marqués de Valdecilla, Santander, Spain; Institute of Parasitology and Biomedicine López-Neyra, CSIC, Granada, Spain

## Abstract

**Introduction:**

A study was conducted to determine whether the human leukocyte antigen (HLA) B alleles are implicated in the susceptibility to Henoch-Schönlein purpura (HSP) in the largest series of Caucasian HSP patients ever assessed for genetic studies.

**Methods:**

The study population was composed of 349 Spanish patients diagnosed with HSP fulfilling the American College of Rheumatology and the Michel *et al.* classification criteria, and 335 sex and ethnically matched controls. *HLA-B* phenotypes were determined by sequencing-based typing (SBT) and analyzed by chi-square or Fisher exact test.

**Results:**

A statistically significant increase of HLA-B*41:02 allele in HSP patients when compared with controls was found (8.3% versus 1.5% respectively; P = 0.0001; OR (odds ratio) =5.76 [2.15-19.3]). These results remained statistically significant after adjusting for Bonferroni correction (P = 0.0028). An internal validation also confirmed the susceptibility effect on HSP associated with HLA-B*41:02 (OR = 5.70 [1.98-16.44]). Since a former study described an association between HLA-DRB1*01:03 and HSP susceptibility, we also evaluated the implication of HLA-B*41:02 independently of HLA-DRB1*01:03. Interestingly, the association remained statistically significant (P = 0.0004, OR = 4.97 [1.8-16.9]). No *HLA-B* association with specific HSP clinical features was found.

**Conclusions:**

Our study indicates that HLA-B*41:02 is associated with the susceptibility to HSP in Spanish patients irrespective of *HLA-DRB1* status.

## Introduction

Henoch-Schönlein purpura (HSP), also called immunoglobulin-A (IgA) vasculitis, is a leukocytoclastic vasculitis characterized by IgA dominant immune deposits involving mainly the skin but other tissues as well. HSP is more common in children but it is not exceptional in adults [1]. The main feature of this vasculitis is a palpable purpura involving predominantly the lower extremities. Besides skin involvement, HSP often causes joint pain and gastrointestinal complications [2]. Renal manifestations may also be observed in HSP patients, mainly in adults, indicating a poor prognosis of this disorder [2].

HSP has a multifactorial pathology in which a wide variety of pathogens, drugs, and other environmental exposures have been involved [3]. Furthermore, several studies have revealed the relevant role of some genetic variants (including those located in the human leukocyte antigen (HLA) system [4]) in both susceptibility and HSP clinical heterogeneity.

The HLA region includes a group of genes located in chromosome 6 (6p21) that encodes for proteins on the surface of cells that are responsible for regulation of the immune system in humans [5]. HLA has been described as a common genetic component that underlies immune-mediated diseases [6], being associated with more diseases than any other region of the human genome [5]. In this regard, the association between class II *HLA* genes and HSP susceptibility in Caucasians has been well-established in a recent well-powered study [4]. In this work, the susceptibility effect of HLA-DRB1*01 (mainly due to HLA-DRB1*01:03 allele) was confirmed, whereas a potential protective effect of HLA-DRB1*03 (mainly due to HLA-DRB1*03:01 allele) was also postulated [4]. However, the influence of class I *HLA* genes in HSP Caucasian patients still remains unclear since only a few studies, performed in small cohorts of HSP patients, have addressed this issue [7-10]. Although no association between class I *HLA* (*HLA-A*, *HLA-B*, and *HLA-C*) genes and HSP was found by Ostergaard *et al.* [10], HLA-B*35 allele has been suggested to be a potential marker of renal complications secondary to HSP by Nathwani *et al.*, Nyulassy *et al.* and Amoli *et al.* [7-9].

Taken together these considerations prompted us to investigate the potential implication of *HLA-B* gene in the susceptibility to HSP. For this purpose we took advantage of the largest series of Caucasian patients with this vasculitis ever assessed for genetic studies.

## Methods

### Patients and study protocol

A series of 349 Spanish patients with cutaneous vasculitis who fulfilled Michel *et al.* [11]*.* classification criteria for HSP were included in the present study. According to these criteria, they were classified as having HSP if they fulfilled three or more of the following characteristics: palpable purpura, bowel angina, gastrointestinal bleeding, macroscopic or microscopic hematuria, age at disease onset ≤20 years, and no previous history of medications prior to the onset of the disease. Also, all patients included in this series were required to fulfill the American College of Rheumatology classification criteria for HSP [12]. Blood samples were obtained from patients recruited from Hospital Universitario Lucus Augusti (Lugo), Hospital Universitario Marqués de Valdecilla (Santander), Hospital Universitario La Princesa (Madrid), Hospital Universitario San Cecilio (Granada), Hospital Universitario Virgen del Rocío (Sevilla) and Hospital Universitario de Basurto (Bilbao). Information on the main clinical features of the whole series of 349 HSP Spanish patients recruited in this study is shown in Table 1. Hematuria with or without proteinuria and severe gastrointestinal manifestations were frequently observed in these patients. However, only 24 of the 349 patients (6.8%) had persistent renal involvement (renal sequelae) at last follow-up.

**Table 1:** **Main clinical features of a series of 349 Spanish patients with HSP**

**Main characteristics**	**% (n/N)**
Children (age ≤20 years)/adults (age >20 years)	283/66
Male/female	176/173
Age at the onset of the disease (years)	
mean ± SD	14.8 ± 18.0
median (IQR)	7 (5 to 18)
Duration of follow-up (years, mean ± SD)	3.3 ± 4.3
Palpable purpura and/or maculopapular rash	100 (349/349)
Arthralgia and/or arthritis	56.4 (197/349)
Gastrointestinal manifestations (if ‘a’ and/or ‘b’)	53.6 (187/349)
a) Bowel angina	52.1 (182/349)
b) Gastrointestinal bleeding	16.0 (56/349)
Renal manifestations (if any of the following characteristics)	35.5 (124/349)
a) Hematuria	34.6 (121/349)
b) Proteinuria	32.9 (115/349)
c) Nephrotic syndrome	4.3 (15/349)
d) Renal sequelae (persistent renal involvement)^a^	6.8 (24/349)

^a^At last follow-up. HSP: Henoch-Schönlein purpura; SD: standard deviation; IQR: interquartile range.

A set of 335 sex and ethnically matched controls without history of cutaneous vasculitis or any other autoimmune disease comprising blood donors from the National DNA Bank Repository (Salamanca, Spain), was also included in the study.

A subject’s written consent was obtained according to the declaration of Helsinki, and the study was approved by the Ethics Committees of Galicia for Hospital Universitario Lucus Augusti, of Cantabria for Hospital Universitario Marqués de Valdecilla, of Madrid for Hospital Universitario La Princesa, of Andalucía for Hospital Universitario San Cecilio and Hospital Universitario Virgen del Rocío, and of País Vasco for Hospital Universitario de Basurto.

### Genotyping

High-molecular-weight genomic DNA was extracted from whole blood using the QIAamp DNA Blood Mini Kit (Qiagen, Hilden, Germany) according to the manufacturer’s instructions.

All DNA samples were stored at −20°C until the HLA analysis. Class I *HLA B* locus high-resolution typing was performed through sequencing-based typing (SBT), by using the SBTexcellerator Kit and analyzed with the SBTengine®-SBT HLA typing software (GenDx, Utrecht, The Netherlands) following the manufacturer’s instructions.

Positive controls were used to ensure the quality control for the genotyping procedures. These samples, of known sequence, were obtained from External Proficiency Testing Programs (GECLID – Spanish Society of Immunology), following the European Federation of Immunogenetics standards. Additionally, negative controls were included to discard the effect of DNA contamination.

### Statistical analysis

Continuous data are described as mean and standard deviation (mean ± SD) and categorical variables as percentages.

The strength of association between HSP and *HLA-B* phenotypes was estimated using odds ratios (OR) and 95% confidence intervals (CI). Levels of significance were determined using contingency tables by either the chi-square test or Fisher exact (expected values below 5) analysis. Results were adjusted for Bonferroni correction. To obtain an internal validation, we carried out a bootstrap test with 1,000 replications.

All analyses were performed with STATA statistical software 12/SE (Stata Corp., College Station, TX, USA).

The linkage disequilibrium between HLA alleles was calculated using the PLINK software [13].

## Results

When HSP patients were compared with matched controls, some differences in *HLA-B* phenotype frequencies were observed (Table 2). In this regard, the HLA-B*41 phenotype was significantly increased in HSP patients compared to controls (10.1% *versus* 3.3%, respectively; *P* = 0.0007; OR = 3.18 (1.53 to 7.07)). This association was mainly due to the HLA-B*41:02 allele (8.3% in HSP patients *versus* 1.5% in controls; *P* = 0.0001; OR = 5.76 (2.15 to 19.3)) and remained statistically significant after adjusting for Bonferroni correction (*P* = 0.0028) (Table 2). Since the potential effect of HLA-B*41:02 on HSP had not previously been reported, we carried out a bootstrapping procedure that confirmed the susceptibility effect on HSP associated with HLA-B*41:02 (OR = 5.70 (1.98 to 16.44)). However, no statistically significant results were observed regarding other *HLA-B* phenotypes and HSP susceptibility (Table 2).

**Table 2:** ***HLA-B*** **phenotype frequencies in patients with HSP and controls**

***HLA-B***		**Patients with HSP (number = 349)**	**Controls (number = 335)**	***P***	**OR (95% CI)**
B*07	B*07:02	52 (14.9)	49 (14.6)	0.92	1.02 (0.65 to 1.60)
B*08	B*08:01	29 (8.3)	31 (9.2)	0.66	0.88 (0.50 to 1.56)
B*13	B*13:02	7 (2.0)	8 (2.4)	0.73	0.84 (0.25 to 2.67)
B*14	B*14:01	8 (2.3)	5 (1.5)	0.44	1.54 (0.44 to 6.07)
	B*14:02	29 (8.3)	47 (14.0)	0.017	0.55 (0.33 to 0.93)
B*15	B*15:01	25 (7.2)	22 (6.6)	0.76	1.10 (0.58 to 2.10)
	B*15:17	4 (1.2)	5 (1.5)	0.69	0.76 (0.15 to 3.60)
B*18	B*18:01	36 (10.3)	41 (12.2)	0.42	0.82 (0.49 to 1.36)
B*27	B*27:05	20 (5.9)	20 (6.0)	0.89	0.96 (0.48 to 1.91)
B*35	B*35:01	50 (14.3)	51 (15.2)	0.74	0.93 (0.59 to 1.45)
	B*35:03	20 (5.7)	8 (2.4)	0.027	2.48 (1.02 to 6.61)
B*38	B*38:01	10 (3.0)	27 (8.1)	0.0027	0.34 (0.14 to 0.73)
B*39	B*39:01	8 (2.4)	7 (2.1)	0.85	1.10 (0.34 to 3.61)
B*40	B*40:01	16 (4.6)	17 (5.1)	0.76	0.89 (0.42 to 1.93)
	B*40:02	10 (3.0)	5 (1.5)	0.22	1.94 (0.59 to 7.33)
B*41 ^a^	B*41:01	6 (1.8)	6 (1.8)	0.94	0.95 (0.25 to 3.62)
	B*41:02	**28 (8.3)**	**5 (1.5)**	**0.0001**	**5.76 (2.15 to 19.3)**
B*44	B*44:02	35 (10.0)	16 (4.8)	0.009	2.22 (1.17 to 4.39)
	B*44:03	61 (17.5)	71 (21.2)	0.22	0.79 (0.53 to 1.17)
B*45	B*45:01	7 (2.0)	11 (3.3)	0.29	0.60 (0.19 to 1.73)
B*49	B*49:01	24 (7.2)	34 (10.1)	0.12	0.65 (0.36 to 1.16)
B*50	B*50:01	26 (7.7)	16 (4.8)	0.14	1.60 (0.81 to 3.26)
B*51	B*51:01	53 (15.2)	56 (16.7)	0.59	0.89 (0.58 to 1.37)
B*52	B*52:01	5 (1.5)	11 (3.3)	0.11	0.43 (0.11 to 1.35)
B*53	B*53:01	12 (3.6)	13 (3.9)	0.76	0.88 (0.36 to 2.13)
B*55	B*55:01	4 (1.2)	12 (3.6)	0.035	0.31 (0.07 to 1.04)
B*57	B*57:01	22 (6.3)	12 (3.6)	0.10	1.81 (0.84 to 4.10)
B*58	B*58:01	13 (3.7)	11 (3.3)	0.75	1.14 (0.46 to 2.85)

^a^10.1% in HSP patients *versus* 3.3% in controls; *P* = 0.0007; OR = 3.18 (1.53 to 7.07); values are expressed as number (n) and percentages (%); the result that remained statistically significant after adjusting for Bonferroni correction (p_BNF_ = 0.0028) is highlighted in **bold**. HLA: human leukocyte antigen; HSP: Henoch- Schönlein purpura; OR: odds ratio; CI: confidence interval.

Since in a recent study we disclosed an association between HLA-DRB1*01:03 and HSP susceptibility [4], we also evaluated whether the implication of HLA-B*41:02 was independent of the HLA-DRB1*01:03 status (Table 3). Interestingly, and as shown in Table 3, the association of HLA-B*41:02 with HSP susceptibility remained statistically significant irrespective of HLA-DRB1*01:03 (*P* = 0.0004, OR = 4.97 (1.8 to 16.9)) (Table 3). To further confirm the independence of both alleles, we calculated the linkage disequilibrium between them in our study population. As expected, the r^2^ value showed that both alleles were independent (r^2^ = 0.003).

**Table 3:** HLA-B*41:02 **and** HLA-DRB1*01:03 **are independently associated with increased susceptibility to HSP**

HLA-DRB1*01:03	HLA-B*41:02	**Patients with HSP**	**Controls**	***P***	**OR (95% CI)**
-	-	270	292	-	Ref.
-	+	23	5	0.0004	4.97 (1.8 to 16.9)
+	-	48	6	<0.001	8.65 (3.6 to 25.1)
+	+	1	0	0.2988	-

HLA: human leukocyte antigen; HSP: Henoch- Schönlein purpura; OR: odds ratio; CI: confidence interval.

In a further step we assessed whether *HLA-B* phenotype differences might exist according to specific features of the disease, such as age at disease onset before and after 20 years, presence of joint, gastrointestinal or renal manifestations. In this regard, we did not observe any trend toward significance between different *HLA-B* phenotypes and gastrointestinal manifestations. Regarding age at disease diagnosis, we only found a statistically marginal increase in the frequency of adults (>20 years old) carrying the HLA-B*45:01 allele. However, this marginal significance was lost after Bonferroni correction. A statistically significant increase in HLA-B*14:02 and *53:01* was detected in HSP patients with renal manifestations (*P* = 0.0113 and 0.0415, respectively). However, similarly to the previous case, when we corrected these results by Bonferroni, this significance was lost.

Finally, 12 HSP patients with renal manifestations carried the HLA-B*41:02 allele. However, no statistically significant association was observed between the presence of this *HLA-B* allele and the presence of renal manifestations (*P* = 0.513; OR = 1.31 (0.53 to 3.17)).

## Discussion

The vasculitides constitute a heterogeneous group of diseases that have in common the presence of inflammation of the blood vessels [1,14]. Their complex etiology is far from being completely elucidated. Both environmental and genetic factors appear to influence the development and progression of these conditions, HLA being the main genetic factor related to these diseases. Although the effect of the class II *HLA* genes in the susceptibility of HSP in Caucasians has recently been characterized [4], there is scarce information on the potential implication of class I *HLA* genes in HSP [7-10]. Because of that, we have performed a study to provide evidence of the potential implications of the *HLA-B* gene in the susceptibility to HSP. For this purpose, we recruited the largest series of Caucasian patients with this vasculitis ever assessed for genetic studies. Accordingly, our findings support the role of the HLA-B*41:02 as a susceptibility marker of this disease irrespective of *HLA-DRB1* status. However, in contrast to previous studies [7-9], we could not confirm an association between *HLA-B* alleles and renal manifestations or any other specific features of this vasculitis. In this regard, considering that our analysis was performed in a well-powered cohort of HSP patients, we cannot exclude the possibility that previous results might have been the result of a type I error. Because of that, additional studies including large series of patients are needed to fully establish the association of the *HLA-B* locus with specific features of HSP.

Class I HLA molecules have previously been associated with different types of primary systemic vasculitis in Caucasians. An association of HLA-B*15, HLA-B*8, HLA-Cw3 and *HLA-Cw6* with giant cell arteritis (a large-sized blood vessel vasculitis) has been reported [15-17]. Although the involvement of class I *HLA* genes in Kawasaki disease (a vasculitis involving medium-sized blood vessels) is controversial, HLA-B*51 and HLA-B*44 have been proposed to be associated with this disease [18,19]. Additionally, HLA-B*50 has been shown to be related to susceptibility to granulomatosis with polyangiitis, a small-sized vessel anti-neutrophil cytoplasmic antibody-associated vasculitis [20]. Finally, a recently published analysis of imputed genome wide association study data described an association between a genetic variant located between the *HLA-B* and *MICA* loci and Behçet’s disease [21]. Regarding HLA-B*35:03, other authors reported an increase of this allele in overall HSP [22]. Even if in our study we observed a significantly increased frequency of this allele in our HSP cohort, this significance was lost after Bonferroni correction. However, this lack of significance after correction in our study might be due to sample size or also to differences between our cohort and that studied by Peru *et al*. [22].

## Conclusions

The analysis of our data supports an association of HLA-B*41:02 with susceptibility to HSP in Caucasians, irrespective of *HLA-DRB1* status. These results may have potential clinical implications as they may help to better identify individuals at risk for this vasculitis.

## Abbreviations

CI: confidence interval
HLA: Human Leukocyte Antigen
HSP: Henoch-Schönlein purpura
IgA: immunoglobulin-A
IQR: interquartile range
OR: odds ratio
SBT: sequencing-based typing
SD: standard deviation
